# Loss of *OBSCN* expression promotes bladder cancer progression but enhances the efficacy of PD-L1 inhibitors

**DOI:** 10.1186/s13578-025-01379-w

**Published:** 2025-03-27

**Authors:** Tao Wang, Tuanjie Guo, Juanjuan Sun, Xinyue Zang, Lei Dong, Jian Zhang, Siteng Chen, Guihua Chen, Sicong Ma, Xinyu Zhai, Chuanmin Chu, Chaofu Wang, Xiang Wang, Dongliang Xu, Mingyue Tan

**Affiliations:** 1 Department of Urology, Shuguang Hospital, Shanghai University of Traditional Chinese Medicine, Shanghai, China; 2 Surgical Institute of Integrative Medicine, Shuguang Hospital, Shanghai University of Traditional Chinese Medicine, Shanghai, China; 3 Surgical Institute, Shuguang Hospital, Shanghai University of Traditional Chinese Medicine, Shanghai, China; 4 Department of Urology, Shanghai General Hospital, Shanghai Jiao Tong University School of Medicine, Shanghai, China; 5 Department of Pathology, Shanghai General Hospital, Shanghai Jiao Tong University School of Medicine, Shanghai, China; 6 Department of Pathology, Ruijin Hospital, Shanghai Jiao Tong University School of Medicine, Shanghai, China; 7 Department of Urology, Shanghai Geriatric Medical Center, Shanghai, China; 8 Department of Urology, Renji Hospital, Shanghai Jiao Tong University School of Medicine, Shanghai, China

**Keywords:** Bladder cancer, Immunotherapy, Immune checkpoint inhibitor, *OBSCN*, Obscurin

## Abstract

**Background:**

As the objective overall response rate to immune checkpoint inhibitors (ICIs) is less than 30% in late stage or metastatic bladder cancer (BLCA), elucidating the intrinsic mechanisms of immune evasion is of great importance for the discovery of predictive and prognostic biomarkers and the exploration of novel targets for intervention. Recent studies have shown that *OBSCN* and the cytoskeletal protein it encodes, obscurin, play an important role in tumour progression. However, no studies have reported the role of *OBSCN* in BLCA.

**Methods:**

RNA sequencing and clinical data were downloaded from multiple public databases including The Cancer Genome Atlas and the Gene Expression Omnibus. Immunohistochemistry (IHC) was performed on tissue microarrays including 80 BLCA patients from Shuguang Hospital. Kaplan-Meier curves with log-rank test, univariate and multivariate COX regression were performed to evaluate the prognostic efficacy of *OBSCN* expression. In vitro experiments were conducted to determine the role of *OBSCN* deficiency in promoting BLCA progression. Pan-cancer tumour immune microenvironment (TIME) analysis was performed to explore the potential correlation between *OBSCN* deficiency and immune evasion.

**Results:**

Pan-cancers and single-cell sequencing analysis revealed that the expression level and proportion of *OBSCN* was significantly decreased in BLCA cells compared to normal urothelium. Survival curves showed that BLCA patients with low *OBSCN* expression had a worse prognosis, yet a better clinical response to PD-L1 ICIs. Gene set variation analysis and Gene set enrichment analysis revealed that epithelial-mesenchymal transition (EMT) and immune-related processes were significantly enriched in BLCA samples with low *OBSCN* expression. In vitro experiments identified that *OBSCN*-deficient BLCA cells enhanced invasion, migration and EMT. Pan-cancer analysis of TIME revealed that neoantigen, tumor mutation burden, CD8^+^T cells and immune checkpoints were significantly negatively associated with *OBSCN* expression. IHC and Western blot assay identified that BLCA samples with low OBSCN expression had more CD8^+^ T-cell infiltration and higher PD-L1 expression.

**Conclusions:**

This study confirmed that BLCA patients with low *OBSCN* expression had a worse prognosis but a superior response to ICIs, providing a reference for individualised treatment of BLCA patients.

**Supplementary Information:**

The online version contains supplementary material available at 10.1186/s13578-025-01379-w.

## Introduction

The most recent data indicate that bladder cancer (BLCA) is the fourth most prevalent malignant neoplasm in males, with 20–25% of these cancers exhibiting muscle-invasive and 5% metastasizing to other sites at the time of initial diagnosis [1]. In recent years, immunotherapy has emerged as a promising new treatment option for metastatic BLCA [1, 2]. PD-1/PD-L1 immune checkpoint inhibitors (ICIs) have been approved for maintenance therapy after platinum chemotherapy or as first-line treatment for patients with platinum drug intolerance [1, 2]. Currently, PD-L1 expression, tumor mutation burden (TMB), and microsatellite instability (MSI) have been approved by the U.S. Food and Drug Administration to predict the efficacy of ICIs [1, 3]. Nevertheless, the insufficient prediction accuracy and high measurement technical requirements restrict their extensive clinical application [1, 4]. Further investigation is required to elucidate the mechanism of immune evasion in BLCA and to identify more precise biomarkers to predict the survival and the efficacy of ICIs in BLCA patients.

*OBSCN* is located on human chromosome 1q42.13 and encodes obscurin, a cytoskeletal protein that plays a scaffold and regulatory role in striated muscle [5]. The majority of contemporary research on the structure, regulation, binding, and pathophysiological functions of *OBSCN* and obscurin originates in striated muscle [5]. Due to its molecular diversity and presence in multiple locations of muscle cells, obscurin can be employed as a scaffold during myogenesis and can act as a mechanical sensor [5]. Furthermore, it facilitates cell adhesion and regulates calcium homeostasis [5]. Consequently, *OBSCN* mutations are linked to various forms of skeletal muscle and cardiomyopathy, including rhabdomyolysis, myocardial remodeling, and arrhythmias [6–8]. In recent years, an increasing number of studies have demonstrated that *OBSCN* plays a pivotal role in the development of tumors [5]. In breast and colorectal cancer, *OBSCN* and *TP53* are the only co-mutated genes among more than 13,000 candidate genes [9]. The loss of *OBSCN* expression can induce cytoskeletal remodeling or promote epithelial-mesenchymal transition (EMT), and promote the growth, migration, and metastasis of pancreatic or breast cancer cells [10, 11]. To date, no studies have reported the role of *OBSCN* in BLCA.

In this study, we initially conducted a pan-cancer analysis to investigate the mutation and expression of *OBSCN* in tumors. Subsequently, the study focused on BLCA to determine the predictive role of *OBSCN* expression on BLCA prognosis, as well as its role in BLCA progression. Finally, pan-cancer analysis was performed to assess the impact of *OBSCN* expression on the tumor immune microenvironment (TIME). This was followed by a focus on *OBSCN* expression as a predictor of response to ICIs and the potential mechanism of immune evasion in BLCA.

## Materials and methods

### Patient cohorts and data resources

In this study, the RNA-sequencing and clinical data of patients were downloaded from The Cancer Genome Atlas (TCGA) (https://portal.gdc.cancer.gov/) and the Gene Expression Omnibus (GEO) (https://www.ncbi.nlm.nih.gov/geo). This study involves eight published datasets, including TCGA-BLCA (*n* = 404), GSE48075 (*n* = 73), GSE31684 (*n* = 93), GSE32894 (*n* = 224), GSE176307 (*n* = 90), E-MTAB-4321 (*n* = 476), IMVigor210 (*n* = 348), and a single-cell sequencing database, GSE135337 (*n* = 5). Besides, the prognostic role of obscurin was further verified in a Shuguang cohort (*n* = 80). We retrospectively recruited 80 patients with BLCA who underwent surgical treatment at the Shuguang Hospital Affiliated to Shanghai University of Traditional Chinese Medicine from September 2010 to December 2015. The GTEX database (https://commonfund.nih.gov/GTEx) was also used to download the gene expression data of different organizations and calculate the gene expression difference among different types of cancer after combining with TCGA data. The Cancer Cell Line Encyclopedia (CCLE) database (https://portals.broadinstitute.org/ccle/) was explored to analyze the expression levels of *OBSCN* among tumor cell lines.

### Genetic alteration analysis

cBioPortal (https://www.cbioportal.org/) database was utilized to evaluate the alteration frequency and mutation site of *OBSCN* in pan-cancers [12]. Besides, SNP-related data of BLCA in TCGA database was downloaded, and top 30 genes with high mutation frequency were selected as display. The differences of mutant genes in BLCA patients with low and high *OBSCN* expression were compared, and mutation landscape maps were drawn with R package ComplexHeatmap.

### Analysis of single-cell RNA-sequencing data

**Fig. 1:**
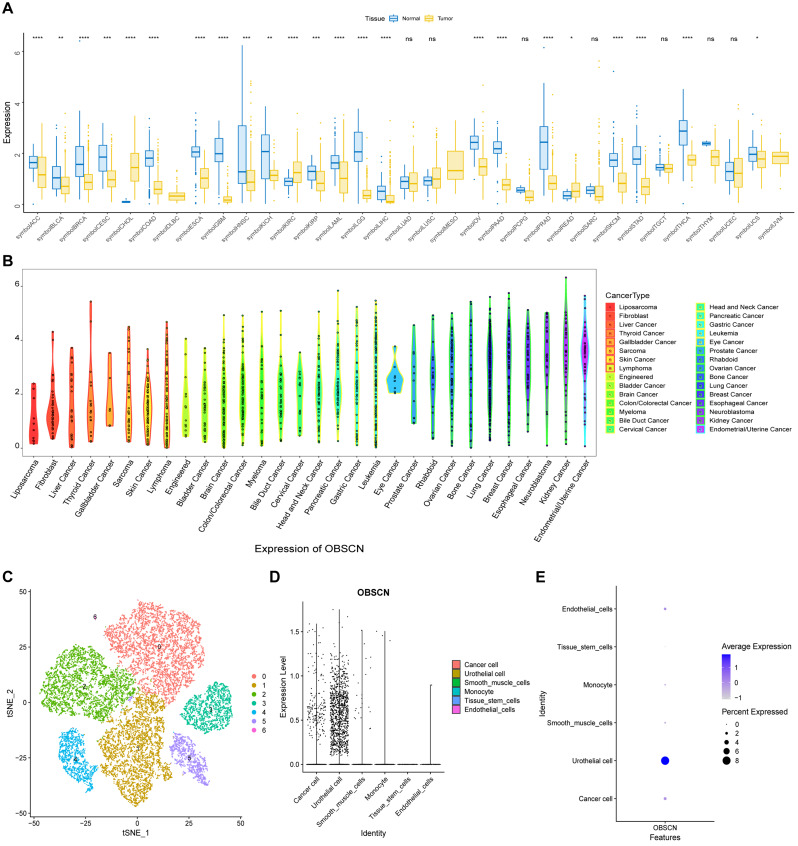
*OBSCN* expression in pan-cancer analysis. (**A**) The expression of *OBSCN* in tumors and adjacent tissues were revealed in pan-cancer analysis; (**B**) Pan-cancer analysis of *OBSCN* expression in tumor cell lines; (**C**) tSNE degradation and visualization analysis of *OBSCN* expression in single-cell sequencing database GSE135337; (**D**–**E**) Expression levels and proportions of *OBSCN* among six types of cells in TIME of BLCA annotated with R package SingleR

Single-cell data of GSE135337 was used in this analysis. GSE135337 contained seven BLCA samples and one paracancerous sample. Only cells with nFeature_RNA > 1000&percent.mt < 5&nCount_RNA < 100,000 in the expression profile were retained for this analysis. The feature expression levels of 23,011 cells were included for subsequent analysis. Data were standardized and homogenized and combined to reduce technical deviations. In this study, 2000 highly variable genes were selected for PCA dimensionality reduction in order to reduce the dimensions of the data, while retaining the most important variation information. Harmony was further used to analyze dimensionality reduction and batch removal. Based on this optimal number of PCS, this study applied the t-distributed stochastic neighborhood embedding (tSNE) method to further reduce and visualize the data, and obtained 11 subtypes by tSNE. In this study, each subtype was annotated using the R-package SingleR, eleven clusters were annotated into six cell categories, including cancer cells, urothelial cells, smooth muscle cells, monocyte, tissue stem cells, endothelial cells (Fig. 1C). The study also investigated the expression of *OBSCN* in these types of cells (Fig. 1D-E).

### Immunohistochemical detection and interpretation of obscurin protein expression

The expression of obscurin protein was detected by IHC. Rabbit anti-human OBSCN antibody was purchased from Proteintech Company (55281-1-AP). Rabbit anti-human obscurin monoclonal antibody were diluted at 1:100 ratio. CD8A Rabbit antibody (A11856) and PD-L1 Rabbit antibody (A1645) were obtained from Abclonal at a dilution ratio of 1:100. IHC staining EnVision method was used, the specific operation steps were carried out according to the kit instructions, and the primary antibody was replaced by PBS as the negative control. The staining intensity of cytoplasm was evaluated by H-score semi-quantitative method. The average H-score of cancer-containing tissue points was calculated for each case, and the patients were divided into H-score = 0 and H-score > 0 groups. H-score of 0 is negative, meaning that obscurin protein is missing. H-score > 0 is considered positive. Blind interpretation was performed by 2 pathologists in the field of urologic tumors according to the above scoring criteria. Besides, CD8^+^T cell counts were enumerated as the mean value of 3 randomized high power magnification fields (HPF, 200× magnification) of each sample. The percentage of stained viable tumor cells over total tumor cells (tumor proportion score, TPS) was calculated for PD-L1 staining [13].

### Pathways and function enrichment analysis

In this study, gene sets were downloaded from Molecular signatures database (v7.0), and each gene set was comprehensively scored using Gene set variation analysis (GSVA) algorithm to evaluate potential biological functional changes in different samples [14]. Besides, “clusterprofiler” and “enrichplot” kits were used to perform Gene set enrichment analysis (GSEA) analysis and compare the differences in signaling pathways between groups with high or low *OBSCN* expression [15].

### Cell culture and Si-RNA transfection

Human BLCA cell lines UMUC3 and 5637 were obtained from American Type Culture Collection (ATCC). All cells were cultured in RPMI 1640 medium (Gibco) supplemented with 10% fetal bovine serum (FBS, Gibco) and 1% penicillin and streptomycin (Gibco). 3.75 µL of Lipofectamine3000 reagent (Invitrogen) and 250 pmol siRNA (Tsingke) were separately added to 125 µL of serum-free, opti-MEM (Gibco) media, and mixed gently. Finally, the mixed solution was added to the cells cultured in 1000 µL fresh RPMI 1640 medium with 10% FBS after 15 min incubation at room temperature. Subsequent experiments were carried out after 48 h of continuous cell culture. The sense sequences of siRNA are as follows siOBSCN#1: 5’-AGAGGCAGGAGCCAGUGCCACACUGAGCU-3’; siOBSCN#2: 5’-CGGAGGUGAUGUGGUACAAAG-3’.

### Western blot assay (WB)

RIPA lysis buffer and a BCA protein quantification kit (Beyotime) were used to extract total protein from the cells and determine protein concentration, respectively. Equal amounts of protein were subjected to sodium dodecyl sulfate polyacrylamide gel electrophoresis and transferred to a membrane. It was incubated with the anti-OBSCN (Cat#A18110, Proteintech, 1:1000) at 4 ◦C overnight after blocking for 1 h at room temperature. The membrane was washed and incubated with horseradish peroxidase-conjugated secondary antibody at room temperature for 1 h. Finally, protein bands were detected using an ECL kit (Beyotime) and protein expression levels were analyzed. Additional antibody information is as follows: anti-β-actin (Cat#AC038, ABclonal, 1:20000), anti-Vimentin (Cat#A19607, ABclonal, 1:1000), anti-Snail (Cat#A11974, ABclonal, 1:1000), anti-N-Cadherin (Cat#A19038, ABclonal, 1:1000), anti-E-Cadherin (Cat#A22333, ABclonal, 1:1000), anti-Slug (Cat#A1057, ABclonal, 1:1000), anti-PD-L1 (Cat#A19135, ABclonal, 1:1000) and HRP Goat Anti-Rabbit IgG (Cat#AS014, ABclonal, 1:20000).

### Cell proliferation experiment

Cell counting kit-8 (CCK-8, Beyotime) was used to evaluate cell proliferation. Transfected cells were seeded in 96-well plates (2000 cells/well) for the CCK-8 assay. CCK-8 reagent was added to the plates at 0, 24, 48, and 72 after inoculation, and absorbance at 450 nm was measured using a spectrophotometer.

### Wound healing assay

The transfected cells were seeded in a 6-well plate at 100% density, and a straight line was made with the tip of a 200 µL pipette. The fluid in each well was replaced with 2 mL of serum-free RPMI 1640 medium, and the area was photographed at 0 and 24 h. The wound healing ability was measured as the area between the two sides of the wound.

### Transwell assay

First, Transwell cell suspensions were prepared in serum-free RPMI 1640 medium. Then, 2 × 10^4^ cells were inoculated with serum-free RPMI 1640 medium in the upper chamber of a transwell with an aperture of 8 mm, coated without matrix gel for cell migration or with matrix gel for the cell invasion assay, and complete medium containing 10% FBS was added to the lower chamber. Finally, the cotton swab was used to remove the upper chamber cells, while other cells were fixed and stained with 0.1% crystal violet after incubated in 37 ◦C incubator for 48 h. The number of migrating or invading cells was counted under a microscope.

### Pan-cancer analysis of tumor immune microenvironment

TMB is defined as the total number of somatic gene coding errors, base substitutions, insertions, or deletions detected per million bases. This study defined TMB by calculating the frequency of variation and variance/exon length for each tumor sample by dividing the non-synonymous mutation site by the total length of the protein coding region. The MSI values for each TCGA patient were derived from previously published studies [16]. NetMHCpan v3.0 was used to evaluate Neoantigen (NEO) in each patient [17]. Fmsb, limma and dplyr packages were used to analyze the correlation between the expression of *OBSCN* and TMB, NEO and MSI in TIME. The potential relationship between *OBSCN* expression and TIME was explored through the TISIDB website [18]. The correlation between *OBSCN* and the infiltration level of CD8^+^T cells was further analyzed using the TIMER2.0 database (http://timer.cistrome.org) [19].

### Statistical analysis

All statistical analyses were performed in GraphPad Prism 9 software and R language (version 4.2.2). Chi squared test and logistic regression were utilized to analyze the correlation between *OBSCN* expression and clinical features in BLCA. Kaplan-Meier with log-rank test and Cox regression analyses were used to evaluate the prognostic value of *OBSCN* expression. All statistical tests were two-sided, and *p* < 0.05 was considered statistically significant.

## Results

### Genetic changes in *OBSCN* in pan-cancers were revealed through comprehensive analysis

According to the cBioPortal website, *OBSCN* is mutated in 10% of all cancers (Supplementary Fig. 1A). Further investigation of the genetic variation of *OBSCN* in different tumor types in the TCGA dataset revealed that BLCA had the 7th highest frequency of genetic variation, mainly mutation, amplification and depth deletion (supplementary Fig. 1A-B). In addition, missense mutations at 1260 amino acids were found to be the dominant type of genetic variation in all TCGA tumor samples (Supplemental Fig. 2A). By using the cBioPortal website to analyze the mutation frequency of *OBSCN* genes, it was found that several genes, including *TTN*, *TP53*, *MUC16*, *PIK3CA*, were mutated more frequently in samples with *OBSCN* mutations (supplementary Fig. 2B-D). Finally, the expression of *OBSCN* under different gene mutation types and copy number variation was analyzed, and it was found that the expression of *OBSCN* was significantly reduced when the copy number was lost (supplementary Fig. 2E-F).

### Analysis of *OBSCN* expression in pan-cancers

A comprehensive analysis of *OBSCN* expression in 33 types of human cancers, conducted using the TCGA combined GTEx dataset, revealed a notable reduction in the expression of *OBSCN* in the majority of tumors, including BLCA (Fig. 1A). Figure 1B illustrated the expression of *OBSCN* in different tumor cell lines as observed in the CCLE. Single-cell sequencing analysis further demonstrated that the expression levels and proportions of *OBSCN* in BLCA cells were significantly decreased in comparison to normal urothelium (Fig. 1C-E).

### Correlation between *OBSCN* expression and clinical characteristics of BLCA

**Fig. 2:**
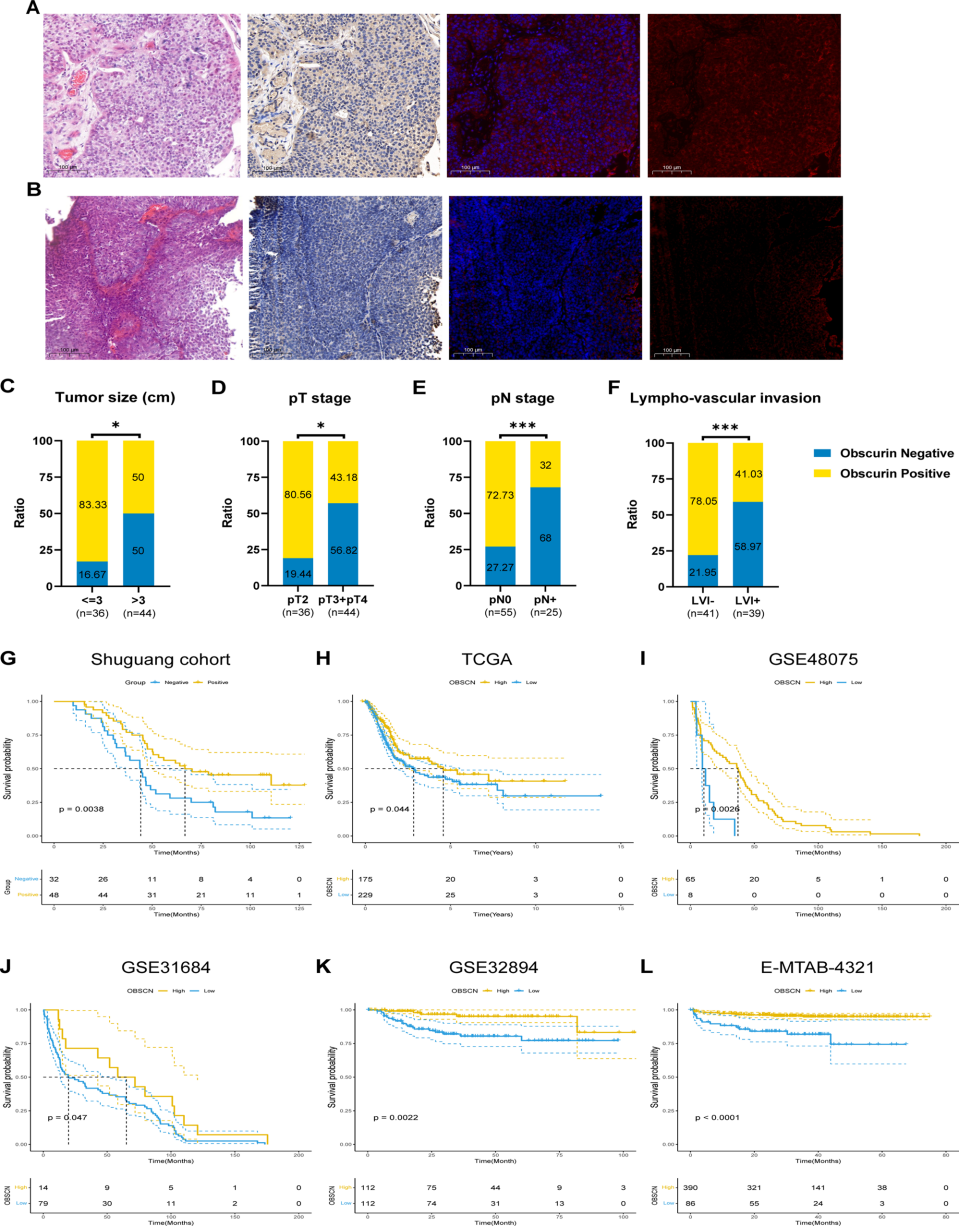
Relationship between *OBSCN* expression and clinical parameters and OS of BLCA. (**A**) HE, IHC and immunofluorescence staining of obscurin in low grade BLCA; (**B**) HE, IHC and immunofluorescence staining of obscurin in high grade BLCA; (**C**–**F**) Relationship between *OBSCN* expression and tumour size, pT stage, pN stage, and lympho-vascular of Shuguang cohort; (**G**) The relationship between *OBSCN* expression and OS in the Shuguang cohort; (**H**) The relationship between *OBSCN* expression and OS in the TCGA cohort; (**I**) The relationship between *OBSCN* expression and OS in the GSE48075 cohort; (**J**) The relationship between *OBSCN* expression and OS in the GSE31684 cohort; (**K**) The relationship between *OBSCN* expression and OS in the GSE32894 cohort; (**L**) The relationship between *OBSCN* expression and OS in the E-MTAB-4321 cohort

Subsequently, IHC staining of obscurin was performed on the tissue microarrays (TMAs) of the Shuguang cohort (Fig. 2 A-B). Chi squared and univariate logistic regression analysis both demonstrated that the absence of *OBSCN* expression was significantly associated with tumor size, pT stage, pN stage, and lymph vascular invasion (Fig. 2 C-F, supplementary Tables 1–2). Further multivariate logistic regression analysis indicated that *OBSCN* expression was significantly associated with pT stage (T2 vs. T3-4 OR = 0.215, *P* = 0.009) (Supplementary Table 2). To further identify the correlation between *OBSCN* expression and clinical parameters, we explored two public databases, TCGA and e-MTAB-4321, which mainly included muscle-invasive bladder cancer (MIBC) and non-muscle invasive bladder cancer (NMIBC), respectively. In TCGA, it was confirmed that *OBSCN* expression was associated with grade, pT stage, and pN stage (Supplementary Tables 3–4). While in e-MTAB-4321 database, it was revealed that *OBSCN* expression was associated with age, grade, and pT stage (Supplementary Tables 5–6). Multivariate logistic regression analysis showed that *OBSCN* expression were significantly correlated with grade and pT stage in both databases (Supplementary Tables 4,6).

### The prognosis role of *OBSCN* expression in BLCA

In view of the close correlation between *OBSCN* expression and clinical characteristics of BLCA, we investigated its potential prognostic role. It was found that BLCA patients with negative *OBSCN* expression exhibited a significantly worse overall survival (OS) and recurrence-free survival (Fig. 2G, supplementary Fig. 3). Univariate COX regression analyses showed that maximum tumour size, pT stage, pN stage, pM stage, lympho-vascular invasion, nerve invasion, and *OBSCN* expression were associated with worse OS (Table 1). In multivariate COX regression analyses, it was demonstrated that pM stage, nerve invasion and *OBSCN* expression were independent predictors of BLCA prognosis (Table 1). To verify this conclusion, we further investigated BLCA cohorts from multiple public databases, including TCGA, GSE48075, GSE31684, GSE894, E-MTAB-4321. It was also found that BLCA patients with low *OBSCN* expression exhibited worse OS through Kaplan-meier with log-rank tests (Fig. 2H-L).

**Table 1:** Univariate and multivariate Cox regression for predicting OS of BLCA in Shuguang cohort

Clinical	Univariate analysis		Multivariate analysis	
Characteristics	HR (95%CI)	*P*-value	HR (95%CI)	*P*-value
Age				
( < = 65 vs. >65)	1.042(0.605–1.796)	0.881		
Smoker				
(No vs. Yes)	1.009(0.572–1.777)	0.976		
Maximum tumour size (cm)				
( < = 3 vs. >3)	**1.942(1.019–3.702)**	**0.044**	0.737(0.353–1.540)	0.417
Pathologic T stage				
(T2 vs. T3-T4)	**3.866(2.080–7.186)**	**< 0.0001**	1.288(0.534–3.110)	0.573
Pathologic N stage				
(N0 vs. N+)	**1.600(0.929–2.756)**	**0.09**	**0.470(0.201-1.100)**	**0.082**
Pathologic M stage				
(M0 vs. M1)	**6.108(3.363–11.094)**	**< 0.0001**	**6.619(2.887–15.173)**	**< 0.0001**
Lympho-vascular invasion				
(No vs. Yes)	**1.641(0.950–2.835)**	**0.076**	0.827(0.371–1.842)	0.642
Nerve invasion				
(No vs. Yes)	**3.090(1.785–5.349)**	**< 0.0001**	**2.390(1.215–4.701)**	**0.012**
*OBSCN* expression				
(Negative vs. Positive)	**0.461(0.269–0.789)**	**0.005**	**0.457(0.226–0.922)**	**0.029**

BLCA, bladder cancer; OS, overall survival; HR, hazard ratio; CI, confidence interval

The bold font meant that the *p*-value was less than 0.1

While in TCGA database, *OBSCN* expression were correlated with worse OS in univariate COX regression analyses, it acted as an independently predictor of PFS through univariate and multivariate COX regression analyses with E-MTAB-4321 cohort (Supplementary Tables 7–8).

### Deficiency of *OBSCN* expression promoted the progression of BLCA

**Fig. 3:**
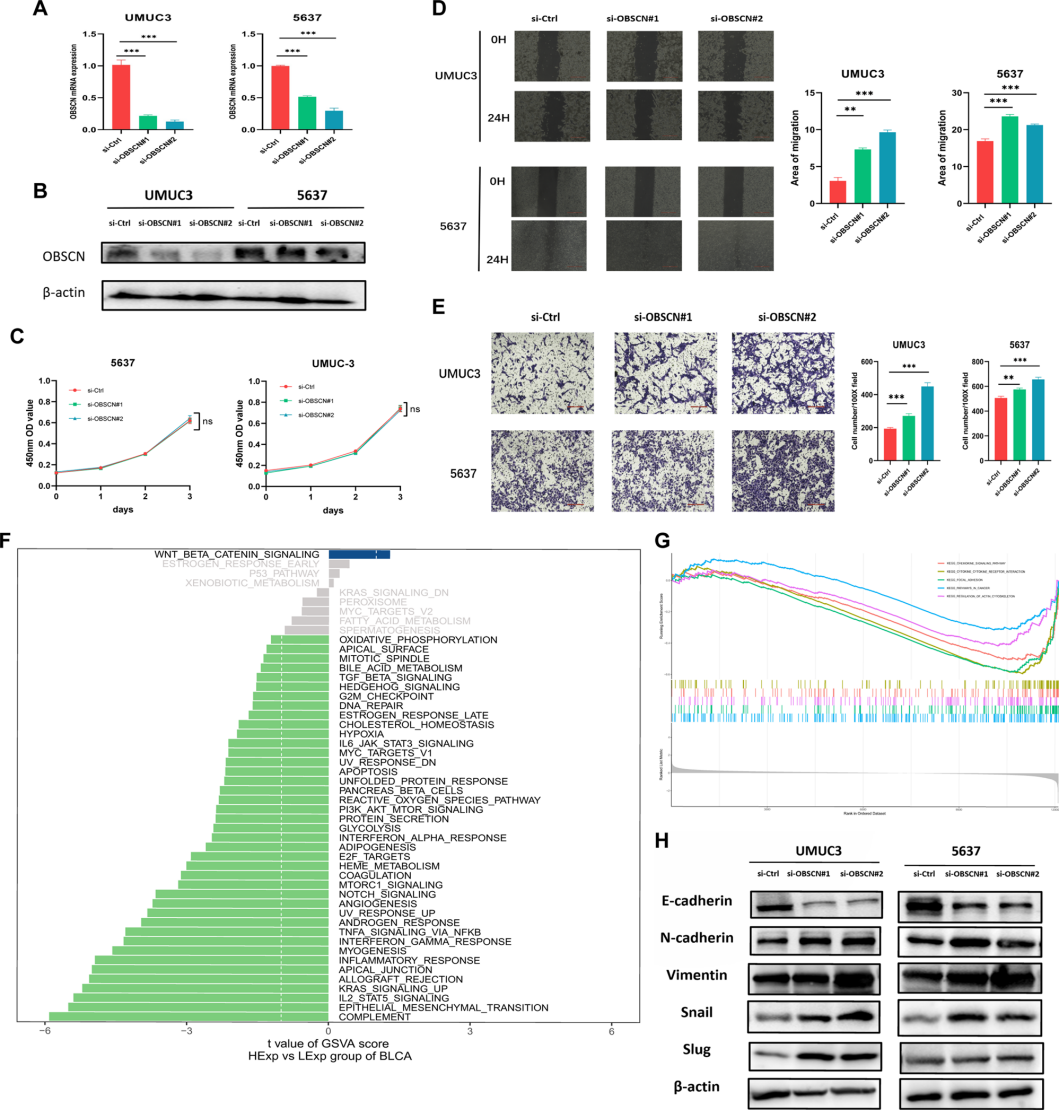
*OBSCN* deficiency promoted the progression of BLCA. (**A**) The mRNA expression of *OBSCN* was stably down-regulated after knockdown; (**B**) The protein expression of *OBSCN* was stably down-regulated after knockdown; (**C**) CCK8 experiment: There was no effect on the proliferative ability of BLCA cells after *OBSCN* knockdown; (**D**) Scratch test: The invasion ability of BLCA cells was enhanced after *OBSCN* knockdown; (**E**) Transwell experiment: The migration ability of BLCA cells was enhanced after *OBSCN* knockdown; (**F**) The results of pathways enrichment were analyzed by GSVA between low and high *OBSCN* expression of BLCA samples; (**G**) The results of pathways enrichment were analyzed by GSEA between low and high *OBSCN* expression of BLCA samples; (**H**) *OBSCN* knockdown enhanced the EMT of BLCA cells

To understand the role of *OBSCN* expression deficiency in the progression of BLCA, we firstly established BLCA cell lines UMUC-3 and 5637 with *OBSCN* knockdown (Fig. 3A-B). In vitro experiments demonstrated that *OBSCN* deficiency significantly enhanced the invasion and migration, while having no effect on the proliferation ability of BLCA cells (Fig. 3C-E). In order to elucidate the underlying mechanism, we initially scored GSVA for BLCA and subsequently divided the samples into high and low expression groups according to the median value of *OBSCN* expression. The results demonstrated that Wnt/beta-Catenin signaling pathway was significantly enriched in the high *OBSCN* expression group, while pathways such as EMT, Kras signaling up were significantly enriched in the group with low *OBSCN* expression (Fig. 3F). Besides, the complement, IL-2-STAT5 signaling, allograft rejection and inflammatory rejection were also significantly enriched in the samples with low *OBSCN* expression, indicating that the absence of *OBSCN* expression may be associated with immune response (Fig. 3F). Furthermore, the chemokine signaling pathway, cytokine-cytokine receptor interaction, and focal adhesion pathways in cancer were also significantly enriched in the *OBSCN* low expression group through the GSEA analysis (Fig. 3G). This further suggested that the absence of *OBSCN* expression was closely related to immune activation and cell motility. Subsequent in vitro experiments demonstrated that the expression of E-cadherin in BLCA cells with *OBSCN* expression deficiency was significantly reduced, while the expression of N-cadherin, Vimentin, Snail, and Slug were significantly increased, indicating that *OBSCN* expression deficiency enhanced the ability of EMT in BLCA cells (Fig. 3H).

### Pan-cancer analysis of the correlation between *OBSCN* expression and TIME

**Fig. 4:**
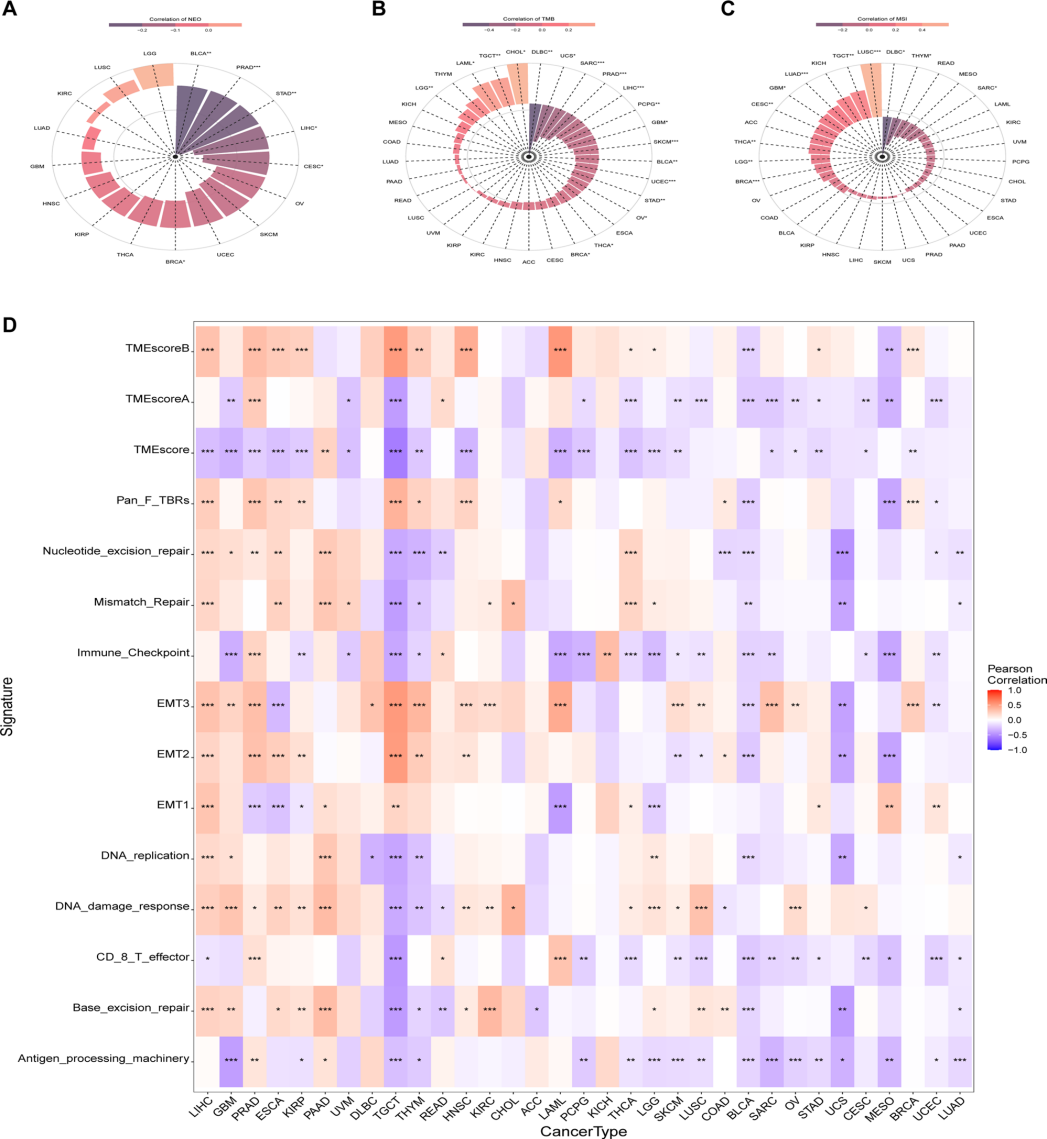
Correlation between *OBSCN* expression and TIME. (**A**–**C**). The relationship between *OBSCN* expression and NEO, TMB, and MSI in pan-cancer analysis; (**D**). Correlation between *OBSCN* expression and essential factors of TIME in pan-cancer analysis

In light of the potential immunoregulatory impact of *OBSCN* expression deficiency on the TIME of BLCA, as indicated by the enrichment analysis, we initially conducted a pan-cancer investigation into the correlation between *OBSCN* expression and NEO, TMB, and MSI, which are emerging biomarkers associated with the efficacy of immunotherapy. The results demonstrated that the expression levels of *OBSCN* were significantly negatively associated with NEO and TMB in several types of tumors, including BLCA (Fig. 4A-B). In the context of MSI, no significant difference was identified in BLCA (Fig. 4C). To further analyze the relationship between *OBSCN* expression and BLCA mutations, we utilized the sequencing mutation data from the TCGA database. Our findings indicated that *TP53*, *TTN*, and other genes exhibited higher mutation frequencies in BLCA with low *OBSCN* expression (Supplementary Fig. 4).

The TIME is primarily comprised of cancer cells, immune cells, tumor-related fibroblasts, extracellular matrix, various growth factors, inflammatory factors, and special physicochemical characteristics [20]. Cancer cell-intrinsic features, including genetic aberrations, signaling pathway deregulation and altered metabolism, play a key role in orchestrating the composition and functional state of the immune landscape and influence the therapeutic response of immunotherapy [20, 21]. Our findings revealed that unlike other types of cancers, the expression of *OBSCN* was significantly negatively correlated with important parameters in the TIME of BLCA, such as TMEscoreB, TMEscoreA, Pan_F_TBRs, nucleotide_excision_repair, mismatch repair, immune checkpoint, EMT3, EMT2, DNA replication, DNA damage response, CD8^+^T effector, base excision repair, and antigen processing machinery, indicating the essential role of *OBSCN* expression deficiency in the immunoregulatory of BLCA (Fig. 4D).

### Predictive value of *OBSCN* expression for the efficacy of ICIs in BLCA

**Fig. 5:**
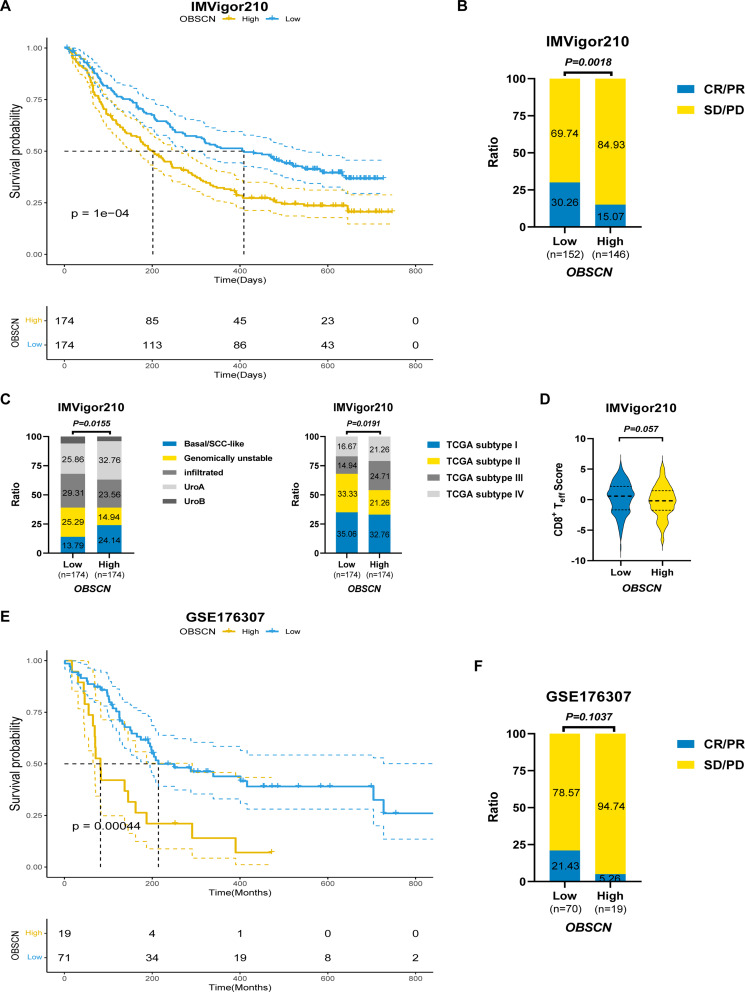
*OBSCN* expression predicted the efficacy of PD-L1 ICIs in BLCA. (**A**). The relationship between *OBSCN* expression and OS in the IMVigor210 cohort; (**B**). Correlation between *OBSCN* expression and clinical response in the IMVigor210 cohort; (**C**). Correlation between *OBSCN* expression and molecular subtypes in the IMVigor210 cohort; (**D**). Correlation between *OBSCN* expression and CD8^+^T effector score in IMVigor210 cohort; (**E**). The relationship between *OBSCN* expression and OS in the GSE176307 cohort; (**A**) Correlation between *OBSCN* expression and clinical response in the GSE176307 cohort

According to the results of the enrichment and TIME analysis, it can be postulated that *OBSCN* expression may affect the efficacy of PD-L1/PD-1 ICIs. By analyzing the IMVigor210 cohort, we unexpectedly found that BLCA patients with low *OBSCN* expression exhibited a higher response to PD-1/PD-L1 ICIs and a better OS (Fig. 5A-B). Concurrently, the molecular subtype distribution of BLCA patients with high or low *OBSCN* expression exhibited notable discrepancies (Fig. 5C). A greater proportion of BLCA samples with low *OBSCN* expression exhibited genomically unstable and TCGA type II characteristics, and higher CD8^+^T effector scores, findings that align with previous TIME analysis (Fig. 5C-D). To further substantiate this conclusion, we conducted a verification analysis in the BLCA immunotherapy cohort GSE176307. Similar findings demonstrated that BLCA patients with low *OBSCN* expression exhibited a superior response to ICIs and a significantly improved OS (Fig. 5E-F).

### Potential mechanisms of *OBSCN* expression deficiency in enhancing the efficacy of ICIs

**Fig. 6:**
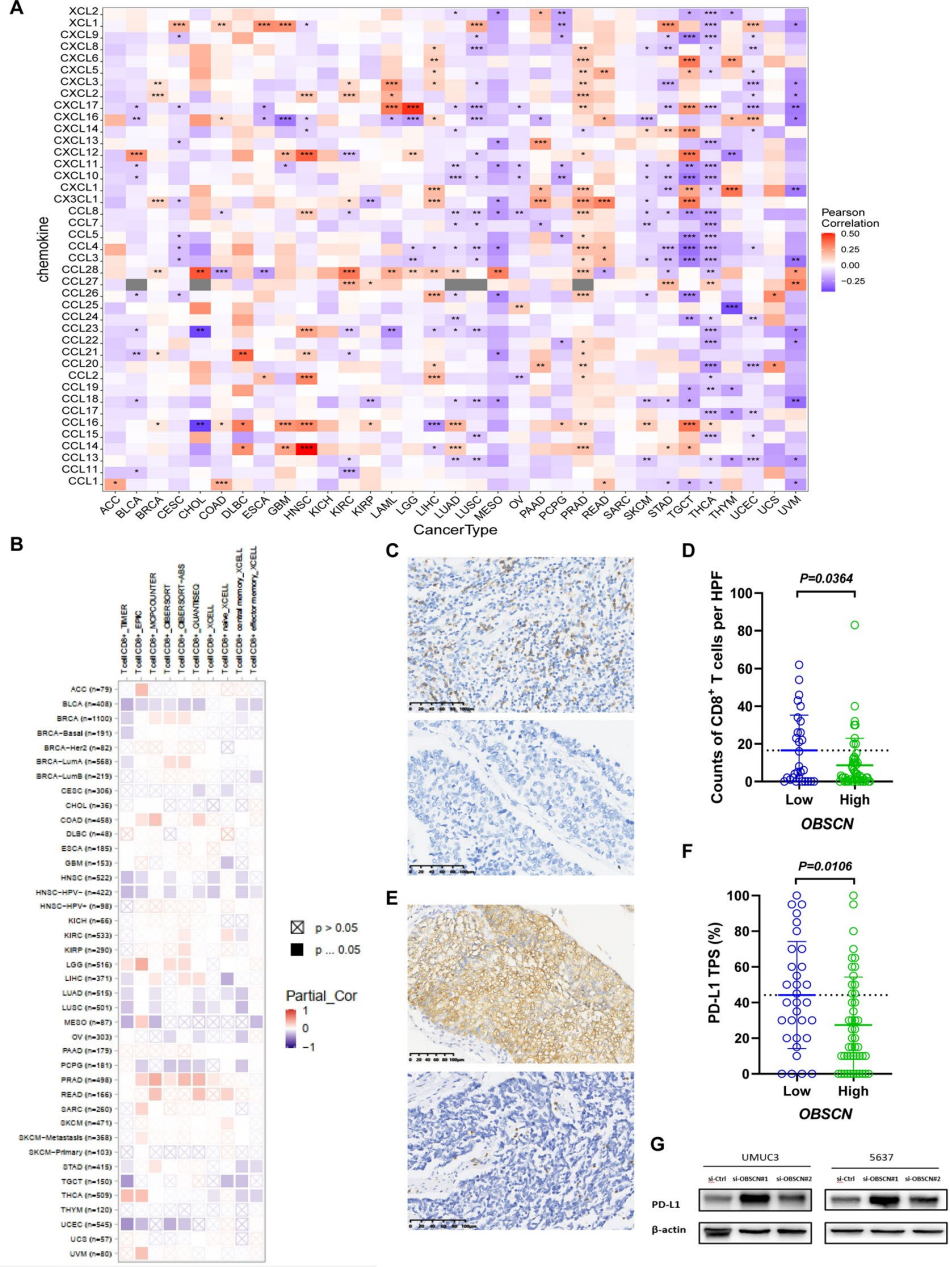
Potential mechanism of *OBSCN* expression deficiency in the immune evasion of BLCA. (**A**) Heatmap representing the correlation between *OBSCN* expression and chemokines in pan-cancer analysis. (**B**) Heatmap representing the correlation between *OBSCN* expression and infiltration of CD8^+^T cells in pan-cancer analysis from TIMER2.0 website. (**C**) IHC staining of CD8A on the TMAs of Shuguang cohort. (**D**) IHC analysis of the CD8^+^T cells between *OBSCN* low and high group in Shuguang TMAs (*n* = 80). (**E**) IHC staining of PD-L1 on the TMAs of Shuguang cohort. (**F**) IHC analysis of the PD-L1 expression in BLCA between *OBSCN* low and high group in Shuguang TMAs (*n* = 80). (**G**) WB test was used to evaluate the expression of PD-L1 after knocking down *OBSCN* in BLCA cell lines UMUC3 and 5637

A preliminary analysis of the TIME and PD-1/PD-L1 therapeutic reactivity in the BLCA patients indicated that *OBSCN* expression deficiency may induce the BLCA to recruit more CD8^+^T cells. The possible reason for worse OS in BLCA patients with low *OBSCN* expression may be the functional exhaustion of CD8^+^T cells owing to the interaction of PD-L1/PD-1 axis between BLCA and CD8^+^T cells, which can be reactivated by using PD-1/PD-L1 ICIs. To identify this hypothesis, we initially performed a pan-cancer analysis of the correlation between *OBSCN* expression and chemokines. The results revealed a significantly negative correlation between *OBSCN* expression and a variety of chemokines that can recruit CD8^+^T cells in BLCA, such as CXCL10 and CXCL11 (Fig. 6A). The significant negative correlation between *OBSCN* and CD8^+^T cells was further identified through TIMER 2.0 database, which includes multiple immune cell infiltration analysis algorithms, such as TIMER, EPIC, MCP-COUNTER, Cibersort, and Xcell (Fig. 6B). Furthermore, we conducted IHC staining for CD8^+^T cells and PD-L1 with the Shuguang cohort, which confirmed that theinfiltration of CD8^+^T cells and expression of PD-L1 in BLCA increased significantly with low *OBSCN* expression (Fig. 6C-F). WB analysis further identified that PD-L1 expression was significantly enhanced in BLCA cells with *OBSCN* knockdown (Fig. 6G).

## Discussion

This study revealed that *OBSCN* expression was deficient in the majority of tumors, including BLCA. Through survival analysis in multiple public databases and our own cohort, it was found that BLCA patients with low *OBSCN* expression exhibited shorter OS. In vitro cell experiments confirmed that the invasion and migration of BLCA with *OBSCN* knockdown were significantly enhanced, as well as the ability of EMT. The results of pathway enrichment and TIME analysis, in vitro experiments and self-owned validation cohort demonstrated that *OBSCN* expression deficiency exhibited significantly increased PD-L1 expression and CD8^+^ T cell infiltration. Two public immunotherapy cohorts demonstrated that BLCA patients with low *OBSCN* expression exhibited a superior response to PD-L1 ICIs.

There was mounting evidence that *OBSCN* was associated with the susceptibility and progression of several types of tumors [9–11, 22]. Among more than 13,000 candidate genes, *OBSCN* and *TP53* are the only commonly mutated genes in breast and colorectal cancer [9]. In pancreatic cancer, Tuntithavornwat et al. demonstrated that pancreatic cancer cells with *OBSCN* expression loss induced cytoskeletal remodeling through downregulation of RhoA activity, thus accelerating cell migration, enhancing motility, and EMT [11]. In vivo studies have demonstrated that loss of *OBSCN* function was associated with increased tumor growth and metastasis [11]. A comparable phenomenon has been documented in breast cancer [10]. In the context of carcinogenic KRAS signaling, the loss of *OBSCN* expression in normal breast epithelial cells has been observed to result in a number of adverse effects, including increased cell survival and chemotherapy resistance, cytoskeleton changes, enhanced cell migration and invasion, and promotion of metastasis [10]. Activation of *OBSCN*-AS1 by CRISPR in triple-negative breast cancer can effectively restore the expression of *OBSCN*, inhibit the migration, invasion, and metastasis of tumor cells in vitro, and may become a therapeutic target for triple-negative breast cancer [22]. Functional and pathway enrichment analysis in our study demonstrated that *OBSCN* expression deficiency was significantly enriched in EMT, and the KRAS signaling pathway was up-regulated. In vitro cell experiments further demonstrated that BLCA with *OBSCN* expression deficiency exhibited enhanced invasion, migration, and EMT.

This study revealed that while BLCA patients with low *OBSCN* expression exhibited a worse OS, they exhibited a superior response to PD-L1 ICIs. Through pan-cancer TIME analysis and self-contained cohort validation, we found that, in contrast to other cancers, *OBSCN* expression in BLCA was significantly negatively correlated with multiple immune-related factors, including CD8^+^T cell infiltration, immune checkpoint expression, EMT score, and TME score. Pan-cancer analysis of the relationship between *OBSCN* expression and chemokines revealed a significant negative correlation between *OBSCN* expression and a variety of chemokines, including CXCL10, CXCL11, CCL11, CCL21, and CCL23. The recruitment of CD8^+^ T cells by chemokines has been demonstrated in multiple studies to occur within the tumor microenvironment [23–26]. For instance, Kumagai et al. demonstrated that RHOA wild-type gastric cancer cells secrete greater quantities of CXCL10 and CXCL11, thereby recruiting more CD8^+^T cells [23]. RHOA mutated gastric cancer cells reduced the secretion of CXCL10 and CXCL11, and stimulated the synthesis of fatty acids through the PI3K-AKT-mTOR pathway, which in turn enhances the inhibition of regulatory T cells (Tregs) within the TIME, thereby inducing immunosuppression [23]. Qi et al. demonstrated that CCL11 was the most effective chemokine in improving immunogenicity, promoting specific CD8^+^T cell infiltration, and inducing tumor rejection [25]. The combination of CCL11 with the E6/E7 antigen as a therapeutic DNA vaccine has the potential to markedly enhance the therapeutic efficacy [25]. In human breast cancer, higher CCL21 expression levels are associated with increased infiltration of CD8^+^T cells and reduced long-term recurrence rates [27]. In ovarian cancer, CCL23, produced by macrophages, was involved in the formation of an immunosuppressive TIME in ovarian cancer by inducing the depletion of T cell phenotypes [28]. The specific chemokine expression induced by the *OBSCN* expression deficiency in BLCA warranted further confirmation in vitro and in vivo.

This study is subject to several shortcomings. Firstly, due to the limited number of patients in the self-verified cohort, it is necessary to expand the cohort to verify the predictive value of *OBSCN* expression loss on the OS and ICIs response of BLCA patients. Secondly, further in vivo experimentation is required to confirm that *OBSCN* expression deficiency may promote epithelial mesenchymal transformation and metastasis of BLCA. Finally, through pan-cancer bioinformatics analysis and self-contained cohort analysis, we discovered that BLCA with *OBSCN* expression deficiency can up-regulate the expression of PD-L1 and secrete more chemokines that recruit CD8^+^ T cells. However, the underlying mechanism remains to be confirmed.

## Conclusions

Our study confirmed that BLCA patients with low *OBSCN* expression had a worse prognosis, yet a superior response to PD-L1 ICIs. This study provided a reference for individualized and precise treatment of BLCA patients.

## Electronic supplementary material

Below is the link to the electronic supplementary material.

Supplementary Material 1

Supplementary Material 2

Supplementary Material 3

Supplementary Material 4

Supplementary Material 5

Supplementary Material 6

Supplementary Material 7

Supplementary Material 8

Supplementary Material 9

Supplementary Material 10

Supplementary Material 11

Supplementary Material 12

Supplementary Material 13

## Data Availability

All data generated that are relevant to the results presented in this article are included in this article. The datasets presented in this study can be found in online repositories. The names of the repository/repositories and accession number(s) can be found in the article/supplementary material.

## Abbreviations

BLCA: Bladder cancer
CCLE: Cancer Cell Line Encyclopedia
EMT: Epithelial-mesenchymal transition
FDA: Food and Drug Administration
GEO: Gene Expression Omnibus
GSEA: Gene set enrichment analysis
GSVA: Gene set variation analysis
ICIs: Immune checkpoint inhibitors
IHC: Immunohistochemistry
MIBC: Muscle-invasive bladder cancer
MSI: Microsatellite instability
NMIBC: Non-muscle invasive bladder cancer
OS: Overall survival
TCGA: The Cancer Genome Atlas
TIME: Tumor immune microenvironment
TMB: Tumor mutation burden
tSNE: t-distributed stochastic neighborhood embedding
WB: Western blot assay
