# Supplementary figures and images for: Loss of OBSCN expression promotes bladder cancer progression but enhances the efficacy of PD-L1 inhibitors
Source: Cell Biosci. 2025 Mar 27;15:40. doi: 10.1186/s13578-025-01379-w (PMC11948897; doi:10.1186/s13578-025-01379-w)

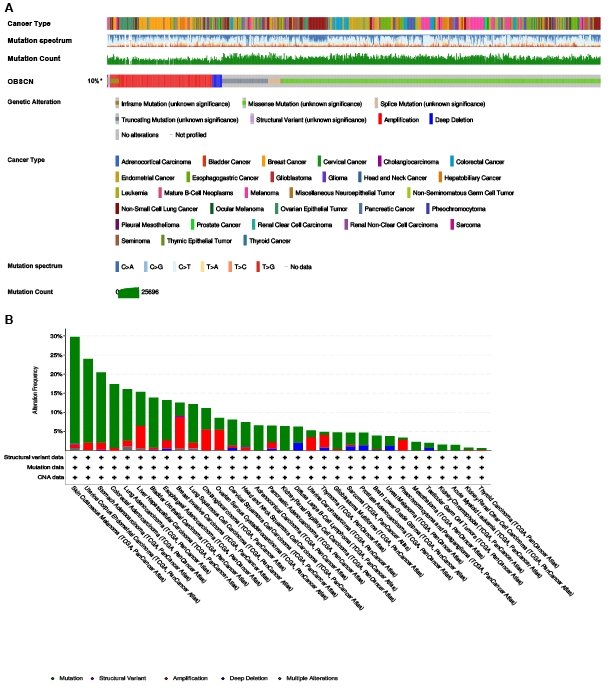

Supplement: Supplementary file 1 — Supplementary Material 1 [file 13578_2025_1379_MOESM1_ESM.png]

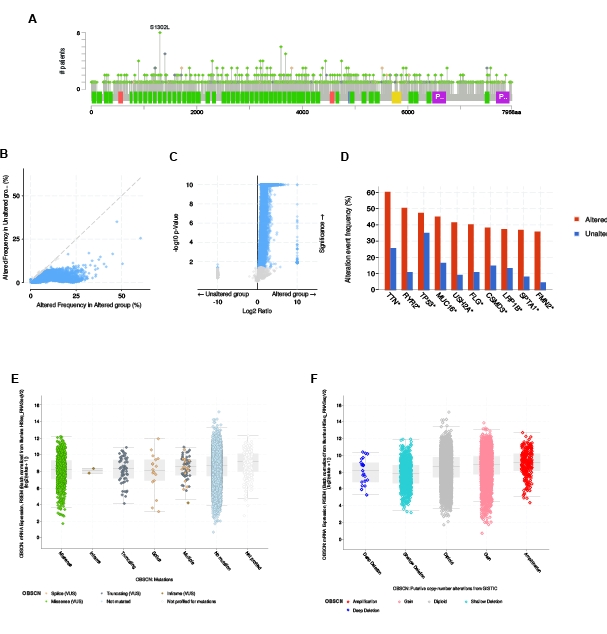

Supplement: Supplementary file 2 — Supplementary Material 2 [file 13578_2025_1379_MOESM2_ESM.png]

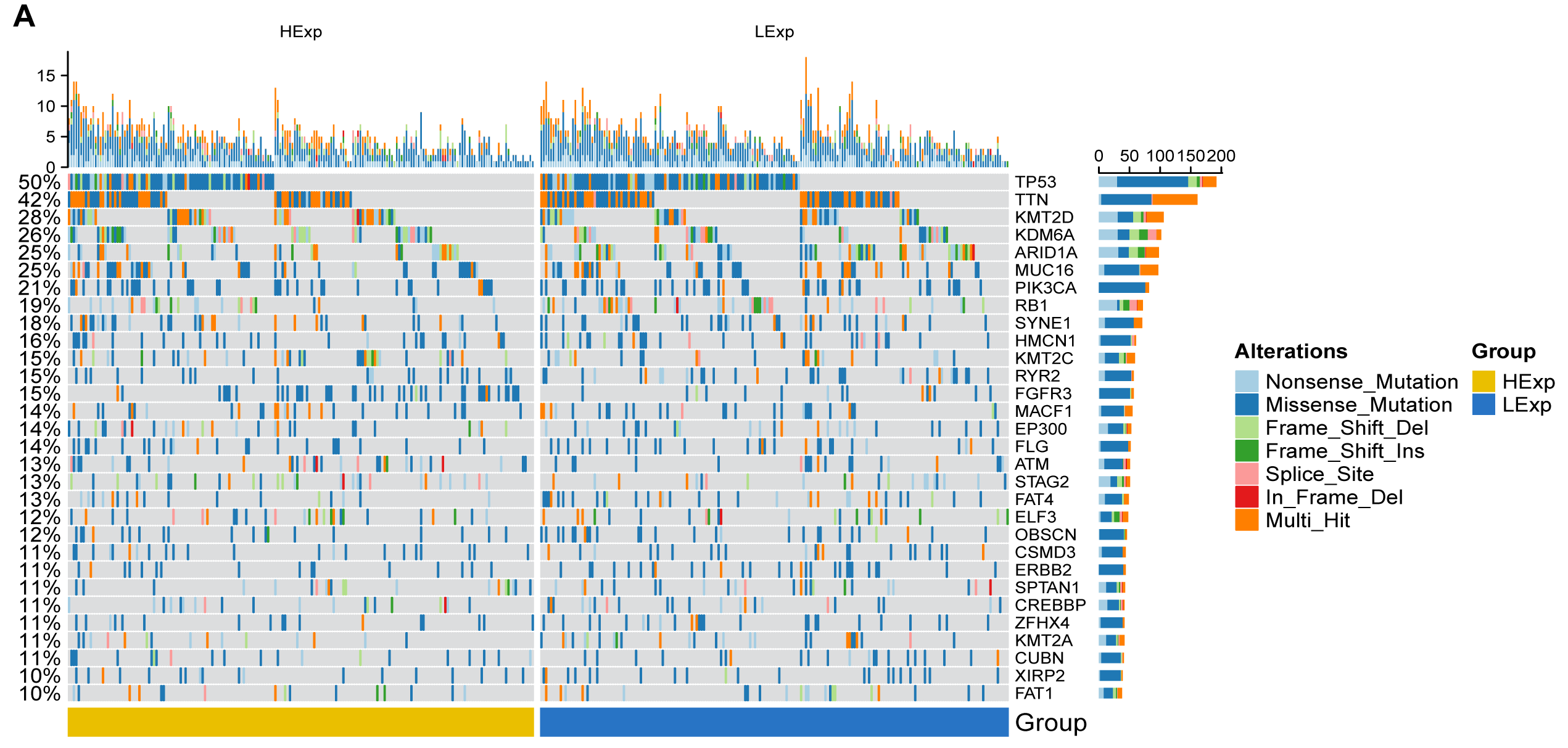

Supplement: Supplementary file 7 — Supplementary Material 7 [file 13578_2025_1379_MOESM7_ESM.tif]

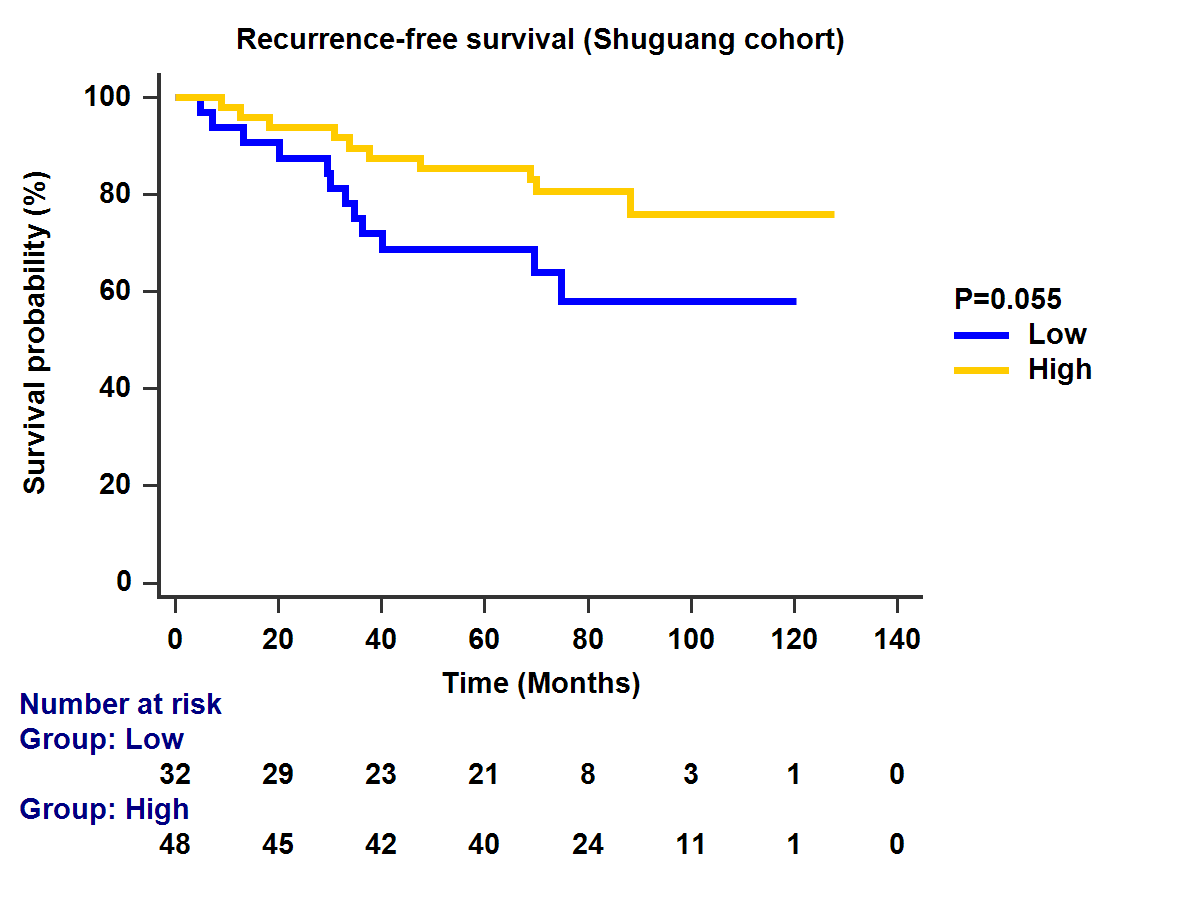

Supplement: Supplementary file 13 — Supplementary Material 13 [file 13578_2025_1379_MOESM13_ESM.tif]
