# Supplementary material for: Loss of OBSCN expression promotes bladder cancer progression but enhances the efficacy of PD-L1 inhibitors
Source: Cell Biosci. 2025 Mar 27;15:40. doi: 10.1186/s13578-025-01379-w (PMC11948897; doi:10.1186/s13578-025-01379-w)
Supplement: Supplementary file 4 — Supplementary Material 4 [file 13578_2025_1379_MOESM4_ESM.docx]

**siRNA**

OBSCN





ACTIN





**EMT**

**UMUC3 AND 5637**

**E-cadherin**


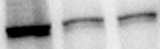


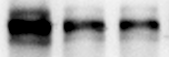


**N-cadherin**


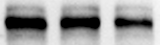


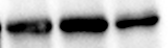


**Vimentin**


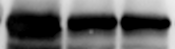


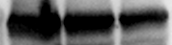


**Snail**


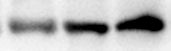


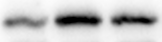


**Slug**


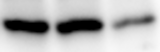


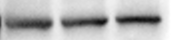


**β-actin**


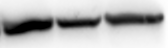


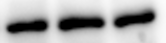


**PD-L1**

PD-L1-5637





PD-L1-UMUC3





ACTIN
